# Lung ultrasound for the diagnosis of pneumonia in adults: a systematic review and meta-analysis

**DOI:** 10.1186/1465-9921-15-50

**Published:** 2014-04-23

**Authors:** Miguel A Chavez, Navid Shams, Laura E Ellington, Neha Naithani, Robert H Gilman, Mark C Steinhoff, Mathuram Santosham, Robert E Black, Carrie Price, Margaret Gross, William Checkley

**Affiliations:** 1 Division of Pulmonary and Critical Care, School of Medicine, Johns Hopkins University, 1800 Orleans Ave Suite 9121, Baltimore, MD 21205, USA; 2 Biomedical Research Unit, A.B. PRISMA, Lima, Peru; 3 Department of International Health, Bloomberg School of Public Health, Johns Hopkins University, Baltimore, MD, USA; 4 Cincinnati Children’s Hospital and Medical Center, Division of Infectious Diseases, Global Health Center, Cincinnati, OH, USA; 5 Welch Medical Library, Johns Hopkins University School of Medicine, Baltimore, MD, USA

**Keywords:** Lung ultrasound, Pneumonia, Meta-analysis

## Abstract

**Background:**

Guidelines do not currently recommend the use of lung ultrasound (LUS) as an alternative to chest X-ray (CXR) or chest computerized tomography (CT) scan for the diagnosis of pneumonia. We conducted a meta-analysis to summarize existing evidence of the diagnostic accuracy of LUS for pneumonia in adults.

**Methods:**

We conducted a systematic search of published studies comparing the diagnostic accuracy of LUS against a referent CXR or chest CT scan and/or clinical criteria for pneumonia in adults aged ≥18 years. Eligible studies were required to have a CXR and/or chest CT scan at the time of evaluation. We manually extracted descriptive and quantitative information from eligible studies, and calculated pooled sensitivity and specificity using the Mantel-Haenszel method and pooled positive and negative likelihood ratios (LR) using the DerSimonian-Laird method. We assessed for heterogeneity using the Q and I^2^ statistics.

**Results:**

Our initial search strategy yielded 2726 articles, of which 45 (1.7%) were manually selected for review and 10 (0.4%) were eligible for analyses. These 10 studies provided a combined sample size of 1172 participants. Six studies enrolled adult patients who were either hospitalized or admitted to Emergency Departments with suspicion of pneumonia and 4 studies enrolled critically-ill adult patients. LUS was performed by highly-skilled sonographers in seven studies, by trained physicians in two, and one did not mention level of training. All studies were conducted in high-income settings. LUS took a maximum of 13 minutes to conduct. Nine studies used a 3.5-5 MHz micro-convex transducer and one used a 5–9 MHz convex probe. Pooled sensitivity and specificity for the diagnosis of pneumonia using LUS were 94% (95% CI, 92%-96%) and 96% (94%-97%), respectively; pooled positive and negative LRs were 16.8 (7.7-37.0) and 0.07 (0.05-0.10), respectively; and, the area-under-the-ROC curve was 0.99 (0.98-0.99).

**Conclusions:**

Our meta-analysis supports that LUS, when conducted by highly-skilled sonographers, performs well for the diagnosis of pneumonia. General practitioners and Emergency Medicine physicians should be encouraged to learn LUS since it appears to be an established diagnostic tool in the hands of experienced physicians.

## Background

Pneumonia is considered a major healthcare and economic problem with a considerable effect on morbidity and mortality worldwide [1-5]. The incidence of community-acquired pneumonia has remained constant over the last few decades affecting 3–5 people per 1000 person-years, predominantly among the young and elderly [6,7]. Even if discharged, patients are still at risk of returning to Emergency Departments (EDs) or clinics and being readmitted with more severe disease [8]. Pneumonia is also an important health-care related complication: it is the second most common type of nosocomial infection and has the highest mortality [9]. Due to this high burden, physicians with patients suspected of pneumonia are constantly challenged to determine if the clinical syndrome is pneumonia rather than alternative diagnosis.

The diagnosis of pneumonia is made by a constellation of suggestive clinical features such as tachypnea, fever, and respiratory rales or reduced breath sounds on auscultation; and, the presence of consolidation or opacification in a chest radiograph (CXR) or in computerized tomography (CT) scan of the chest [10,11]. CXR is the main imaging approach in many settings; however, limitations for its use exist. For example, radiation exposure precludes CXR use in pregnant women [12]. Moreover, it is frequently troublesome to acquire both posteroanterior and laterolateral projections in hospitalized patients [13], especially among the critically-ill. Finally, CXR can be a time consuming procedure and its interpretation has high inter-observer variability among radiologists [14,15]. Chest CT scan, considered the gold-standard imaging approach for pneumonia, has its own limitations: it is expensive; impractical, especially in the critically-ill; and, has higher radiation exposure than CXR [13,16,17].

Use of lung ultrasound (LUS) has long been limited to the diagnosis of pleural effusions, thoracentesis and biopsy-guided procedures; however, it has recently been shown to be highly effective in evaluating pulmonary conditions such as pneumonia and pneumothorax [18,19]. The use of LUS has gained popularity in intensive care units (ICUs) and EDs in the last decade, and has become increasingly recognized as a potentially useful diagnostic approach for community-acquired pneumonia [20-22]. We sought to summarize the existing evidence in published literature and characterize the diagnostic accuracy of LUS for pneumonia in adults.

## Methods

### Search strategy

Two informationists (CP and MG) developed and conducted the search strategy after input from other investigators (MC, NS and WC) in the research team. A systematic literature search was applied to Medline (1946-present). The search was adapted for Embase (1974-Present), Cochrane Library (1898-present), Web of Science (1900-present), Global Health (1973-present) and LILACs (1982-present). We used a combination of controlled vocabulary of keywords around pneumonia and ultrasound (See Search terms for meta-analysis subsection below). We did not limit our search to studies based on publication dates. We did not seek to identify research abstracts from meeting proceedings or unpublished studies as these are not commonly subjected to exhaustive peer-review. Results of the search were reviewed jointly by the research team and the strategy was developed iteratively. We also provided the two informationists with three studies [20-22] that should be included in their search results. We searched all original articles published in English, French or Spanish. All titles and abstracts relevant to our study were retrieved and searched independently by two authors (MC and NS) for full text. References from selected studies and review articles were manually evaluated to identify any possible relevant study for analysis. The literature search and data analysis was performed between June and July 2013.

### Search terms for meta-analysis

(“Pneumonia”[Mesh:noexp] OR “Pneumonia, Bacterial”[Mesh:noexp] OR “Bronchopneumonia”[Mesh] OR “Pleuropneumonia”[Mesh] OR “Severe Acute Respiratory Syndrome”[Mesh] OR “Acute Chest Syndrome”[Mesh] OR “Pneumonia, Aspiration”[Mesh] OR “acute chest syndrome”[tw] OR “acute chest syndromes”[tw] OR “pulmonary inflammation”[tw] OR “pulmonary inflammations”[tw] OR “pulmonary inflammation”[mesh] OR “aspiration pneumonia”[mesh] OR “aspiration pneumonia”[tw] OR “bacterial pneumonia”[tw] OR “bronchiolitis obliterans organizing pneumonia”[tw] OR “bronchopneumonia”[tw] OR “bronchopneumonias”[tw] OR “community acquired pneumonia“[tw] OR “health care associated pneumonia”[tw] OR “hospital acquired pneumonia”[tw] OR “legionnaire disease”[tw] OR “legionnaire s disease”[tw] OR “legionnaires disease”[tw] OR “lobitis”[tw] OR “lung infiltrate”[tw] OR “lung inflammation”[tw] OR “lung inflammation”[tw] OR “Lung Inflammations”[tw] OR “nonspecific inflammatory lung disease”[tw] OR “organizing pneumonia”[tw] OR “peripneumonia”[tw] OR “pleuropneumonia”[tw] OR “pneumonia”[tw] OR “pneumonias”[tw] OR “pneumonic lung”[tw] OR “severe acute respiratory syndrome”[tw] OR “pneumonitis”[tw] OR “lower respiratory tract”[tw] OR “lower respiratory tracts”[tw]) AND (“Ultrasonography”[Mesh:noexp] OR “ultrasonography”[tw] OR “ultrasonographies”[tw] OR “ultrasonic”[tw] OR “ultrasonics”[tw] OR “ultrasound”[tw] OR “ultrasounds”[tw] OR “ultra sound”[tw] OR “ultra sounds”[tw] OR “ultrashell”[tw] OR “ultra shell”[tw] OR “LUS”[tw] OR “sonography”[tw] OR “sonographies”[tw] OR “sonofication”[tw] OR “ultrasonography”[tw] OR “ultrasonographies”[tw] OR “echography”[tw] OR “echographies”[tw] OR “sonogram”[tw] OR “sonograms”[tw] OR “echogram”[tw] OR “echograms”[tw] OR “echoscopy”[tw] OR “echoscopies”[tw] OR “lung ultrasound”[tw] OR”chest ultrasound”[tw] OR “thoracic ultrasound”[tw] OR “lung ultrasounds”[tw] OR “chest ultrasounds”[tw] OR “thoracic ultrasounds”[tw] OR “lung ultrasonography”[tw] OR “lung ultrasonographies”[tw] OR “chest ultrasonography”[tw] OR “chest ultrasonographies”[tw] OR “thoracic ultrasonography”[tw] OR “thoracic ultrasonographies”[tw] OR “lung sonography”[tw] OR “lung sonographies”[tw] OR “chest sonography”[tw] OR “chest sonographies”[tw] OR “thoracic sonography”[tw] OR “thoracic sonographies”[tw] OR “lung echoschopy”[tw] OR 'lung echoscopies”[tw] OR “chest echoscopy”[tw] OR “chest echoscopies”[tw] OR “thoracic echoschopy”[tw] OR “thoracic echoschopies”[tw] OR “lung echogram”[tw] OR “lung echograms”[tw] OR “lung sonogram”[tw] OR “lung sonograms”[tw] OR “chest sonogram”[tw] OR “chest sonograms”[tw] OR “thoracic sonogram”[tw] OR “thoracic sonograms”[tw] OR “lung ultra sound”[tw] OR “chest ultra sound”[tw] OR “thoracic ultra sound”[tw]) NOT (“animals”[mh] NOT (“animals”[mh] AND “humans”[mh])).

### Study eligibility

Inclusion criteria were as follows: enrollment of adult patients aged ≥18 years with clinical suspicion of pneumonia based on respiratory signs and symptoms or acute respiratory failure, and evaluation of pneumonia based on a combination of clinical data, laboratory results and chest imaging by CXR or a chest CT scan. We excluded studies that enrolled children [23,24]. Two investigators (MC and NS) evaluated independently all relevant studies for eligibility criteria and pooled analysis. Data retrieved from these studies by both investigators were compared. We defined *a priori* that disagreements would be achieved via consensus between three members of the study team (MC, NS and WC), which only happened in one study [25] that was eventually excluded after discussion.

### Data extraction

The following data were extracted from each study: sample size; gender proportion or number of adults by gender; mean age; LUS technique; areas evaluated per hemithorax; time lapse between chest imaging and LUS; average time to perform LUS; expertise of operator; blinding; LUS pattern definitions; and, number or proportion of true positives, true negatives, false positives and false negatives. Operator expertise was assessed by the number of LUS procedures performed or by the number of years of LUS experience.

### Methodological quality assessment

Methodological quality was assessed using the QUADAS-2 (Quality Assessment of Diagnostic Accuracy Studies) criterion [26], which provides a standardized approach to grade the quality of studies included in meta-analyses of diagnostic accuracy. QUADAS-2 categorizes the risk of bias and study generalizability as low, unclear or high. Both reviewers (MC and NS) scored the 7-item tool independently and disagreements were resolved via consensus (between MC, NS and WC); i.e., a face-to-face discussion about each disagreement.

### Biostatistical methods

The primary objective was to estimate pooled measurements of diagnostic accuracy: pooled sensitivity and specificity using the Mantel-Haenszel method [27], and pooled positive and negative likelihood ratios (LR) using the DerSimonian-Laird method [28]. We also calculated an overall area under the receiver-operating-characteristic (ROC) curve. Heterogeneity was assessed using the Cochran Q-statistic and the inconsistency (I^2^) test [29]. An I^2^ > 20% was considered as indicative of significant variation [29]. Subgroup sensitivity analyses were also conducted to determine the robustness of findings. We used Meta-DiSc 1.4 [30] and R (http://www.r-project.org) for statistical analyses.

## Results

### Overview of literature search

**Figure 1:**
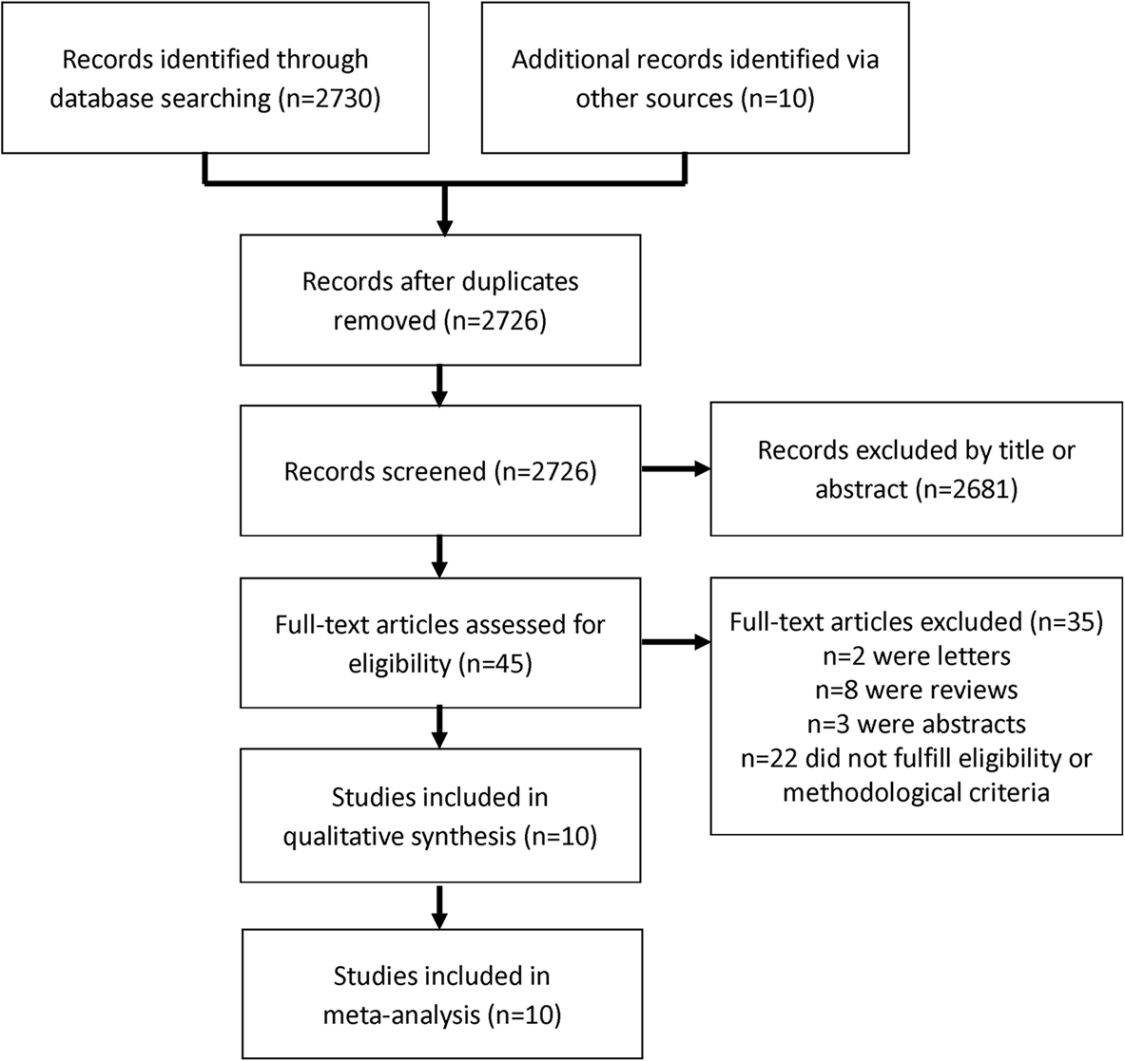
Flowchart of articles retrieved from search of databases and reasons of exclusions.

We identified 2726 studies that fit our search strategy (Figure 1) of which 45 (1.7%) were retrieved for further evaluation based on inclusion criteria and content. After excluding commentaries, review articles, studies not fulfilling methodological criteria and studies involving children, we identified 10 studies [21,22,31-38] for analysis: 6 studies (60%) were conducted in adult patients admitted to EDs or medical wards, and 4 studies (40%) were conducted in adult, critically-ill patients in the ICU. Two studies used lung subunits as independent observations: in one study each patient contributed information for each hemithorax [38] while in another each patient contributed information for twelve lung regions [32].

### Characteristics of selected studies

We describe the main characteristics of eligible studies in Table 1. Mean age was 59 years (range 18 to 95) and 60% were male. Two studies (20%) were conducted in multiple centers [22,36] while the remaining (80%) were single-center studies [21,31-35,37,38]. All studies were blinded to outcome of CXR or chest CT scan prior to conduct or interpretation of LUS. Four studies were performed in Italy [21,31,35,36], three in France [32-34], one in Greece [38], one in [Hong Kong] China [37] and one was a multicenter study in Europe [22]. Only three studies (30%) conducted a follow-up LUS to evaluate resolution of pneumonia [22,31,35]. Four studies (40%) enrolled patients with suspected pneumonia or H1N1 infection who presented to an ED [21,35-37], two studies enrolled hospitalized patients [22,31], and four (40%) enrolled critically-ill patients [32-34,38].

**Table 1:** Characteristics of studies and patients enrolled from studies retrieved for meta-analysis

**Study**	**Year**	**Origin**	**Design**	**Sample size**	**Mean age (years)**	**M/F**	**True positive**	**False positive**	**False negative**	**True negative**
**Benci *et al. *** [31]	1996	Italy	Prospective	57	38.5	50/30	37	0	0	20
**Lichtenstein *et al. *** [32]	2004	France	Prospective	32**	58	Not mentioned	111	0	8	265
**Lichtenstein *et al. *** [33]	2004	France	Prospective	117	53	37/23	59	1	6	51
**Lichtenstein *et al. *** [34]	2008	France	Prospective	260	68	140/120	74	10	9	167
**Parlamento *et al. *** [35]	2009	Italy	Prospective	49	60.9	31/18	31	0	1	17
**Cortellaro *et al. *** [21]	2010	Italy	Prospective	120	69	77/43	80	2	1	37
**Xirouchaki *et al. *** [38]	2011	Greece	Prospective	42*	57.1	34/8	66	4	0	14
**Reissig *et al. *** [22]	2012	Europe	Prospective	356	63.8	228/134	211	3	15	127
**Testa *et al. *** [36]	2012	Italy	Prospective	67	55	Not mentioned	32	5	2	28
**Unluer *et al *** [37]	2013	China	Prospective	72	66.3	35/37	27	7	1	37

**Unit of analysis was 12 lung regions. *Unit of analysis was each hemithorax.

Seven studies (70%) had a highly-skilled physician perform LUS [21,22,31,32,34-36], but only three adequately defined the degree of expertise. Reissig *et al.*[22] considered an expert to be a physician who had performed at least 100 LUS procedures, while two other studies considered an expert to be a physician with more than 10 years of experience in LUS [35,36]. Two studies (20%) trained a general practitioner or ED physician to perform LUS [33,37]. The training approach was explained in only one of the studies, and it consisted of three hours of didactic learning and three hours of hands-on LUS use taught by an experienced radiologist [37]. One study (10%) did not comment on operator expertise [38].

### Methodological heterogeneity

The overall quality of studies included in this meta-analysis was high (Table 2). All studies enrolled patients who probably would have received a CXR or chest CT scan in common clinical practice; described their selection criteria with sufficient detail; and, conducted LUS in a short period before or after chest imaging. Moreover, all studies assessed tests independently and blinded from standard imaging results, and LUS technique were described with sufficient detail. LUS sonographers were not blinded to clinical data. Five studies (50%) used a combination of clinical criteria and imaging as the reference standard (Table 3) [21,22,31,34,36]. Five studies used imaging only as the reference standard: three studies (30%) used chest CT scan for the diagnosis pneumonia in the entire sample [32,33,38] and two (20%) studies used chest CT scan when the results of CXR and LUS were found to be discordant [35,37]. While all studies (100%) obtained a chest CT scan at some point for the diagnosis of pneumonia, only three had it for the entire sample [32,33,38]. Three studies (30%) used chest CT scan as the gold standard in cases for which LUS and CXR findings were discordant [22,31,35] and four (40%) performed chest CT scan when clinically indicated [21,34,36,37]. Four studies (40%) explained reasons for study withdrawal [22,34,36,37] and only one study (10%) reported non-interpretable results [35].

**Table 2:** QUADAS-2 risk of bias assessment

**Study**	**Risk of bias**				**Applicability concerns**		
	**Patient selection**	**Index test**	**Reference standard**	**Flow and timing**	**Patient selection**	**Index test**	**Reference standard**
**Benci *et al. *** [31]	L	?	?	L	L	?	L
**Lichtenstein *et al. *** [32]	L	L	L	L	L	L	L
**Lichtenstein *et al. *** [33]	L	L	L	L	L	L	L
**Lichtenstein *et al. *** [34]	L	L	L	L	L	L	L
**Parlamento *et al. *** [35]	L	L	L	L	L	L	L
**Cortellaro *et al. *** [21]	L	L	L	L	L	L	L
**Xirouchaki *et al. *** [38]	L	L	L	L	L	L	L
**Reissig *et al. *** [22]	L	L	L	L	L	L	L
**Testa *et al. *** [36]	L	L	L	L	L	L	L
**Unluer *et al. *** [37]	L	L	L	L	L	L	L

L: Low risk, ?: Unclear risk, H: High risk.

**Table 3:** Chest imaging and diagnostic criteria of selected studies

**Study**	**Imaging**	**Pneumonia diagnosis**	**Patient type**	**Inclusion criteria**	**Ultrasound operator**	**Diagnostic criteria**	**Blinding**
**Benci *et al. *** [31]	CXR + chest CT if CXR/LUS discordance	Clinical diagnosis or imaging	Hospitalized	Pneumonia symptoms	Experienced physicians	Consolidation	Yes
**Lichtenstein *et al. *** [32]	Chest CT	Imaging only	Critically Ill	Acute respiratory distress syndrome	Experienced physicians	Consolidation	Yes
**Lichtenstein *et al. *** [33]	Chest CT	Imaging only	Critically ill	Chest pain or severe thoracic disease	Two ED physician sonographers	Consolidation	Yes
**Lichtenstein *et al. *** [34]	CXR + chest CT if possible	Clinical diagnosis or imaging	Critically Ill	Acute respiratory failure	Experienced physicians	Alveolar and interstitial	Yes
**Parlamento *et al. *** [35]	CXR + chest CT if CXR/LUS discordance	Imaging only	Presented to ED	CAP symptoms	Experienced physician	Alveolar and interstitial	Yes
**Cortellaro *et al. *** [21]	CXR + chest CT if possible	Clinical diagnosis or imaging	Presented to ED	CAP symptoms	Experienced physicians	Alveolar and interstitial	Yes
**Xirouchaki *et al. *** [38]	Chest CT scan	Imaging only	Critically ill	Mechanically ventilated patients scheduled for chest CT scan	Single physician (Expertise not mentioned)	Consolidation	Yes
**Reissig *et al. *** [22]	CXR + chest CT if CXR/LUS discordance	Clinical diagnosis or Imaging	Presented to ED or hospitalized	CAP symptoms	Experienced physicians	Consolidation	Yes
**Testa *et al. *** [36]	CXR + chest CT if possible/indicated	Clinical diagnosis or imaging	Presented to ED	Suspected H1N1 infection	Experienced physicians	Alveolar and interstitial	Yes
**Unluer *et al. *** [37]	CXR + chest CT if possible/indicated	Imaging only	Presented to ED	CAP symptoms	Trained emergency physicians	Alveolar and interstitial	Yes

*LUS* lung ultrasound, *CXR* chest X ray, *CT* computerized tomography, *ER* emergency department, *ICU* intensive care unit, *CAP* community acquired pneumonia, *US* ultrasound.

Nine studies (90%) used a 3.5-5 MHz micro convex transducer [21,22,31-37] and one (10%) used a 5–9 MHz convex probe [38]. Four studies (33%) provided information regarding time to perform LUS [21,33,36,37] (Table 4). There was no consensus on how to conduct LUS examination across studies: we found substantial heterogeneity on LUS approach and only five studies (50%) examined at least 12 regions of the chest (Table 4). Four studies [21,32,36,37] reported the length of time to conduct LUS. The maximum reported time was 13 minutes.

**Table 4:** Ultrasound characteristics and procedure for assessing the lung

**Study**	**Ultrasound**	**Time of procedure**	**Area examined**
**Benci *et al. *** [31]	Ansaldo AU-560; 3.5 MHz convex probe	Not mentioned	Medio-lateral anterior and posterior intercostal imaging
**Lichtenstein *et al. *** [32]	Hitachi-405; 5-MHz microconvex probe	Less than 3 minutes	2A, 2 L and 2PL
**Lichtenstein *et al. *** [33]	Hitachi Sumi 405, 3.5 MHz micro-convex probe	Not mentioned	Anterior, lateral y posterior
**Lichtenstein *et al. *** [34]	Hitachi-405; 5 MHz microconvex probe	Not mentioned	2A 2 L and 2P
**Parlamento *et al. *** [35]	Megas CVX, Esaote Medical Systems, 3.5- to 5-MHz convex probe	Not mentioned	2A, 2 L and 1P
**Cortellaro *et al. *** [21]	Esaote Medical System; 3.5–5 MHz convex probe	5 min max.minutes maximum	2A, 2 L, 1P. Longitudinal and oblique scans.
**Xirouchaki *et al. *** [38]	Hitachi EUB 8500, 5–9 MHz microconvex probe	Not mentioned	2A 2 L and 2P
**Reissig *et al. *** [22]	Not mentioned; 5 or 3.5 MHz convex probe	Not mentioned	Systematically all intercostal spaces anterior and posterior
**Testa *et al. *** [36]	Toshiba SSA-250A,Esaote MyLab30 and an Esaote Megas CVX; 3 to 6-MHz convex probe	7 to 13 minutes	2A, 2 L and 1P
**Unluer *et al. *** [37]	Mindray Biomedical Electronics Co. M7 model; 3.6-MHz microconvex probe	Less than 10 minutes	2A, 1 L and 1P

Area examined is referred to each hemithorax. Two zones anterior (A): superior and inferior; two lateral (L), and one or two posterior (P).

### Overall meta-analysis

**Figure 2:**
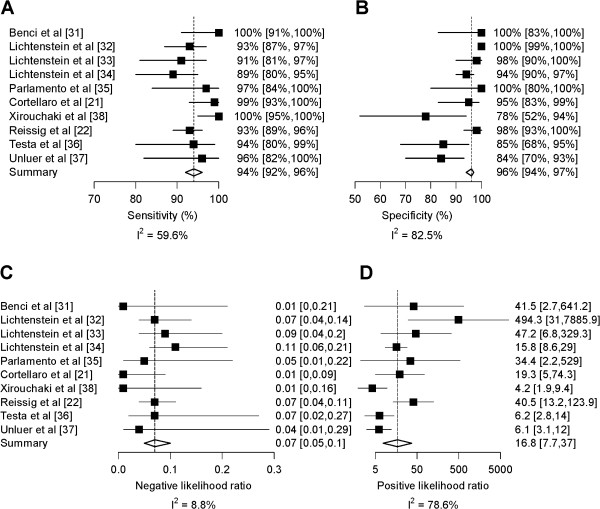
**Forest plots for diagnostic accuracy of lung ultrasound for the diagnosis of pneumonia.** Sensitivity **(Panel A)**, Specificity **(Panel B)**, negative likelihood ratio **(Panel C)** and positive likelihood ratio **(Panel D)**. Inconsistency (I^2^) describes the percentage of total variation across studies due to heterogeneity.

**Figure 3:**
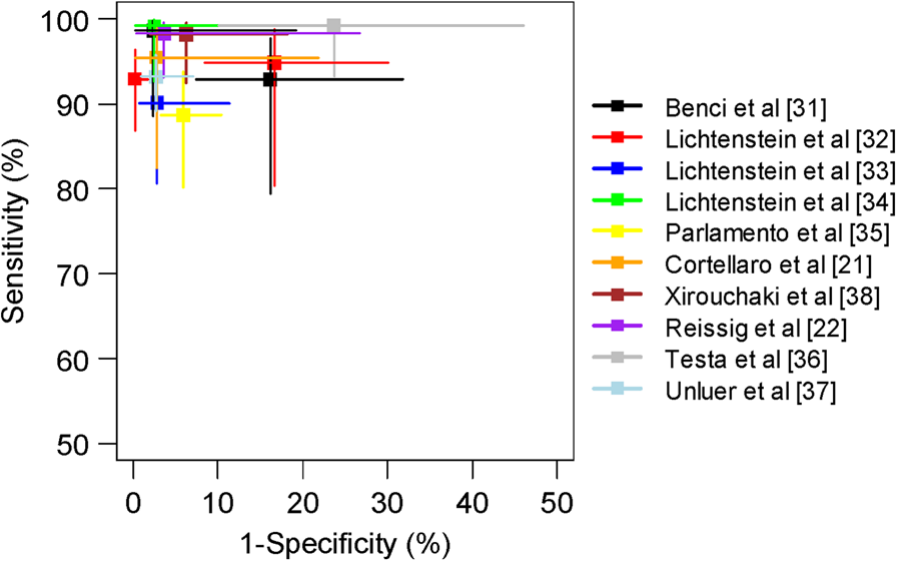
**Summary receiver operating characteristics of lung ultrasound for pneumonia.** The values sensitivity and 1-specificity for each study are represented with a square. 95% confidence intervals for sensitivity (vertical lines) and 1-specificity (horizontal lines) are also shown. Each study is represented by a separate color.

Overall pooled sensitivity and specificity (Figure 2) for the diagnosis of pneumonia were 94% (95% CI, 92% to 96%; p < 0.001) and 96% (95% CI, 94% to 97%; p < 0.001), respectively. The area under the ROC curve was 0.98 (95% CI, 0.98 to 0.99) (Figure 3). Overall pooled positive and negative LRs were 16.8 (95% CI, 7.7 to 37.0; Cochran Q-statistic = 42.0; p < 0.001) and 0.07 (95% CI, 0.05 to 0.10; Cochran Q-statistic = 9.9, p = 0.36), respectively (Figure 2).

### Subgroup analysis by diagnostic imaging criteria

In studies (n = 5) that used the combination of chest imaging and clinical criteria for the diagnosis of pneumonia as the gold-standard, LUS had a pooled sensitivity of 95% (95% CI, 93% to 97%) and a pooled specificity of 94% (95% CI, 92% to 96%) [21,22,31,34,36]. In studies (n = 3) that used chest CT scan only as the gold-standard, LUS had a pooled sensitivity of 94% (95% CI, 90% to 97%) and a pooled specificity of 99% (95% CI, 97% to 100%) [32,33,38]. In studies (n = 5) that used chest imaging alone as the gold-standard, LUS had a pooled sensitivity of 95% (95% CI, 92% to 97%) and pooled specificity of 97% (95% CI, 95% to 98%) [32,33,35,37,38].

A total of 5 studies contributed sufficient information to compare LUS against chest CT scan as the gold standard in individual patients [21,22,32,33,38]. In this subset of 671 patients across the five studies, we found that LUS had a sensitivity of 93% (95% CI, 90% to 96) and specificity of 98% (95% CI, 96% to 99%). Five studies reported diagnostic accuracy for consolidation pneumonia only [22,31-33,38], and in these studies LUS had a pooled sensitivity of 94% (95% CI, 92% to 96%) and pooled specificity of 98% (95% CI, 97% to 99%). An analysis of diagnostic accuracy of LUS for interstitial pneumonia was not possible as no study evaluated interstitial pneumonia alone.

### Subgroup analysis by patient type and physician training

In studies (n = 4) that evaluated critically-ill patients in the ICU, LUS had a pooled sensitivity of 93% (95% CI, 89% to 95%) and pooled specificity of 97% (95% CI, 95% to 98%) [32-34,38]. In studies (n = 6) that evaluated patients admitted to EDs or medical wards, LUS had a pooled sensitivity of 95% (95% CI, 93% to 97%) and pooled specificity of 94% (95% CI, 91% to 97%) [21,22,31,35-37]. In studies (n = 7) in which there was a self-report of expert-level sonographers, LUS had a pooled sensitivity of 94% (95% CI, 92% to 96%) and pooled specificity of 97% (95% CI, 96% to 98%) [21,22,31,32,34-36]. In studies that used ED physicians or general practitioners (n = 2), LUS had a pooled sensitivity of 93% (95% CI, 85% to 97%) and pooled specificity of 92% (95% CI, 84% to 96%) [33,37].

## Discussion

We found that LUS had a high sensitivity (94%) and specificity (96%) for the diagnosis of pneumonia in adults. When we limited our analysis to studies and to an individual patient-level analysis that used chest CT scan alone as the gold standard [21,22,32,33,38], we found a consistently high diagnostic accuracy. LUS performed well both as a rule-in and rule-out test for pneumonia in adults admitted to EDs and medical wards. Even in patients with acute dyspnea, where the differential diagnosis can be broad, LUS had good discrimination. Our meta-analysis supports that LUS, when conducted by highly-skilled sonographers, performs well for the diagnosis of pneumonia. General practitioners and ED physicians should be encouraged to learn LUS for the diagnosis of pneumonia since it is appears to be an established diagnostic tool in the hands of experienced physicians.

Our results differ from that of a contemporary meta-analysis of LUS for the diagnosis of pneumonia that found a sensitivity of 97% and specificity of 94% [39]. There are at least two differences between the meta-analysis conducted by Hu *et al.* and ours. First, Hu *et al.* included studies in both children (n = 5) and adults (n = 4) whereas we limited our analysis to adults only. Studies dealing with different kinds of patient populations may need to be considered separately because they have different gold standards. For example, LUS may perform better in children, which may help explain why Hu *et al.* found a higher sensitivity than we did [23,24]. Second, Hu *et al.* included a fewer number of studies in adults [21,22,35,36]. Specifically, they did not consider two studies of adult patients admitted to EDs or medical wards [31,37] and four studies among critically-ill adults [32-34,38].

Our meta-analyses identified a clear advantage of LUS over standard imaging for pneumonia. Specifically, LUS can be performed in less than 13 minutes. This is substantially shorter than the timeframe required for a CXR or chest CT scan [13,25]. However, there are several limitations that we need to consider when interpreting the evidence in currently published studies. First, not all studies used chest CT scan for the diagnosis of pneumonia as the gold standard. Second, some studies excluded certain populations such as pregnant women [22,35,37], and patients with suspicion of aspiration pneumonia [35], severe immunosuppression [22], interstitial lung disease [34,36,37] and heart failure [36,37]. Third, the protocol for LUS was heterogeneous across studies and this may have affected generalizability. More evidence is needed to provide stronger recommendations in important subgroups.

Our meta-analysis has some shortcomings of its own. First, the total number of studies in our analyses was small; however, this may be offset by the moderate-to-large number of included patients (n = 1172). Second, we did not try to identify studies not published in peer-reviewed journals. Third, only two studies assessed the use of LUS in non-expert physicians who underwent a short training session, precluding our ability to recommend its use in general practitioners. Fourth, all studies were conducted in high-income settings, and none were conducted in resource-poor settings where most of the cases and complications from pneumonia are seen worldwide. Fifth, included studies did not assess all lung regions, as some patients were bedridden and posterior zones were difficult to be assessed.

## Conclusions

LUS has some clear advantages over CXR for patients who are pregnant, bedridden and in resource-limited settings where CXR machines are not currently available. Moreover, it can be done at the bedside, the evaluation is easy and fast to perform and does not suffer from ionizing radiation. Based on our results, LUS is a valid alternative for the diagnosis of pneumonia; however, its role in EDs and in medical wards in the hands of non-expert physicians requires more evidence from well-designed studies.

## Abbreviations

LUS: Lung ultrasound; CXR: Chest X-ray; ICU: Intensive care unit; CT: Computerized tomography; ED: Emergency Department.

## Competing interest

The authors declare that they have no competing interests.

## Authors’ contribution

WC and MC conceived the original study design and were responsible for study conduct. CP and MG conducted public database search based on keywords developed by WC and MC. MC and NS conducted review of published papers and abstraction of data. RG, MSt, MSa, RB provided expert guidance of pneumonia research, analysis and interpretation. LE and NN contributed to writing and interpretation of data. All authors contributed equally to the analysis, interpretation of results, and writing of manuscript. WC had ultimate oversight over study conduct, analysis plan and writing of manuscript. All authors read and approved the final manuscript. Publication of this article was funded in part by the Open Access Promotion Fund of the Johns Hopkins University Libraries. 
